# The Microscope, in Its Application to Practical Medicine

**Published:** 1859-07

**Authors:** 


ART. II.—*The Microscope, in its Application to Practical Medicine.* By LIONEL BEALE, M.B., F.R.S., Physician to King's College Hospital, etc. Second edition. With 270 Wood-cuts, and a Colored Plate. 8vo., pp. 390. London: John Churchill, 1858.

THE prominence assumed by the microscope as a means of medical investigation has of late years led to the issuing of works designed to aid those who would use it, or to set forth the results obtained with it. And each of these objects involves two minor divisions: the former includes the optical principles of the instrument and its accessories, and the various appliances, processes, etc. belonging to the practical pursuit of this branch of study; the latter embraces normal anatomy and pathology.

Each of these four subjects is of great extent and importance; the last two especially have been discussed in works which, voluminous as they are, cannot be called too much so. The microscope must be regarded as an optical instrument; and its employment in medical research merely enables us to see what we could not discover without it. Were it not for the manipulations required in the preparation of specimens, etc. and the various accessory apparatus employed, the only difference actually existing between this instrument and a pair of spectacles would be in the degree of magnifying power. Dr. Beale does not, therefore, mean to treat, as the title of his very handsome manual would lead us to expect, of the microscope, but of its use. In the introduction to his first edition, he disavowed the purpose of showing what the appearances were which the microscope revealed; in the present one he, however, assumes this task, which we think he could hardly hope to accomplish by the addition of only eighty-six pages.

A thorough treatise on the "mode of employment of the microscope in medical investigation" would, we think, be of great value to a very large body of observers; and would not need the somewhat apologetic discussion, nearly eight pages in length, which Dr. Beale has seen fit to prefix to his work. We are referred on page 10, however, to another work by the author for certain matters, the consideration of which he himself says would naturally form the introduction to a work on the use of the microscope; but notwithstanding this disclaimer, the ensuing sixteen pages are devoted to these very matters, with elaborate wood-cuts of some very common and familiar articles. Moreover, many of these illustrations are repeated, in connection with descriptions, in the body of the book.

Here, then, as in fact throughout the entire volume, the reader finds that he has bought an advertisement of the author's other productions, and a number of neat, but useless proofs of certain wood-cuts. We do not see how the fact of an author having previously published another book justifies his omitting what belongs properly to the matter in hand; nor do we like to buy wood-engravings of such things as retort-stands and glass shades. The pages so covered might have been rendered very valuable, had they been occupied by descriptions of the best forms of lamps, and other articles. And though a London class may be very much interested to know that "the German lamps lately introduced by Mr. Pillischer gave an excellent light, and can easily be arranged at any desired height," to the scientific world this fact is of no moment. Again, Dr. Beale's personal friends may be pleased to read, at page 363, that Dr. Saunders some time ago presented him with a beautiful specimen of the *trichina spiralis;* but this item of information will not enable the student to recognize that parasite any more readily. Such Cockneyisms and personalities are out of place in a scientific book.

We must confess also that we are entirely at a loss to appreciate the object of introducing Figs. 86 and 87, in which the human fœtus is depicted of the natural size. Nor can we discover the propriety of devoting three pages to the method of ascertaining the specific gravity of the brain,—a matter entirely foreign to microscopy.

Errors in typography, and especially in punctuation, abound in Dr. Beale's book; but passing over these, we meet with some odd expressions, such as "black vomiting," (p. 366,) "colored situations," (p. 371,) placing objects "in the microscope," and a "pneumonic lung," (p. 202.) Dr. Lawrence Smith is, on page 91, said to reside in *Louisville,* United States, and on page 101 he is transferred to *Louisiana;* an error of some importance to any one desiring to correspond with him. A graver cause of complaint we find in the extreme vagueness of such expressions as "under certain circumstances," (page 203,)—a term which might include any imaginable condition. We are told, on page 228, that "Dr. Billroth, in his investigations, found great advantage in the use of sesquichloride of iron for the purpose of hardening the sections" of the spleen; but are left in doubt whether the sesquichloride was in a solid state or dissolved, and if the latter, in what menstruum, and of what strength. These are just the points which should have been given in full in the work before us. "Brunswick black" is a substance quite frequently alluded to; we are told how to get it off our hands, but not how to make it, or even what it is.

Dr. Beale devotes several pages to directions for drawing and engraving objects. Having some practical acquaintance with this matter, we sincerely pity any beginner who may endeavor to follow out instructions so vague and incomplete,—unnecessary for any one who knows how to draw, and utterly useless to any one who does not.

As we have already remarked, our author assumes the task of briefly discussing normal and pathological anatomy. Our limits will not allow us to do more than point out a few of the defects and superfluities which obscure the real object of the book.

In the first place, the want of a systematic arrangement makes itself apparent to any one seeking information upon a special point. There does not seem to have been, in the author's mind, a clear conception of the exact object of his book,—whether he were treating of comparative anatomy, of manipulation, of organic chemistry, or of pathology. His labors would have been no heavier, while those of the reader would have been much lighter, had the separation of these various subjects been more rigid.

On page 150 is begun a curious discussion of the cell-theory, too vague and brief to be of any use, and yet unsettling to the mind of the beginner, since it hints at changes shortly to be worked in our views upon this subject; such changes will not, however, be effected except by closer reasoning than Dr. Beale is prone to.

At page 157 we are told that the phenomenon of endosmosis depends upon the relative densities of the fluids on either side of a membrane; an idea not now generally received.

The liver is made the subject of a rather long and desultory discussion, with very copious references to our author's other publications, but without any allusion to the valuable researches of Dr. Leidy, published in 1848; the supra-renal capsules, although their consideration occupies six pages, are vaguely treated of, and the only advice given is to examine them in thin section. The directions for investigating the kidney are much better.

Upon the subject of the microscopic study of sputa, very good instructions are given. A pair of fine curved scissors will, however, we think, serve quite as good a purpose as the forceps alluded to on page 271, and are much more likely to be at hand.

Urinary deposits are described at some length, and we think that their microscopic appearances would be most appropriately discussed in a manual like this, although Dr. Beale says "it is not compatible with the objects of this work to do more than give a very short summary of the microscopical characters of the principal urinary deposits, and describe the mode of collecting them and the plans adopted for their preservation."

To discuss the pathological conditions upon which the presence of these deposits may depend, belongs to the wide subject of diseases of the genito-urinary apparatus, and would demand a far larger space. The section on "spermatozoa," (more properly called *spermatozoids*,) might, we think, be condensed to about five lines.

On page 323, after being told that the dumb-bell form of crystal is presented not only by oxalate of lime, but also occasionally by urate of potassa, phosphate of lime, and uric acid, we find a statement that "dumb-bell crystals possess the same chemical characters as the octohedra of oxalate of lime."

Our author's expressions in reference to morbid growths are very vague; he says, for instance, on page 339, that "by simple hypertrophy of the subcutaneous areolar tissue of the leg and foot, or of that of the scrotum, most formidable diseases are caused;" on page 347, "by some the *ovarian tumour* is considered as a form of *colloid*, but, its history and mode of development differ from the gum-like growths met with in other localities." (The italics and punctuation of this sentence are as in the original.)

Fig. 248, on page 341, is said to represent a nucleated fibrous tumor from the tongue; but it has to us very much the appearance of an epithelioma, containing masses of epithelial cells, concentrically arranged and surrounded by a fibrous stroma.

The directions given for the examination of morbid growths are very good, and we particularly agree with Dr. Beale in advising that accurate details of every observation should be entered in a note-book kept for the purpose. Much more space might very well have been devoted to a description of the methods employed for keeping specimens.

Parasites, animal and vegetable, form the subject of the last chapter, but are not fully or practically discussed.

Three years ago, we procured a copy of the first edition of Dr. Beale's book, attracted by its very beautiful appearance, and relying upon the high estimate set upon it by critics. But we have not found it useful as a manual, while it is altogether unsatisfactory as a scientific book. Such subjects as the author admits to be within the scope of his work, he either treats briefly, or refers us for their discussion to some other of his publications; such as he might well exclude, he sets forth vaguely and discursively. No one can hope to bring within the compass of 377 pages even a compendium of practical microscopy, minute anatomy, and material pathology; but when space is wasted in needless illustration and personal compliment, and irrelevant subjects are introduced, when important points are slurred over and trifling ones enlarged upon, the elegant volume resembles a handsome dandy, whose stock in trade consists of his personal appearance and a smattering of small-talk on current topics. Such a man may be very well received, he may even make himself agreeable in general society; but among practical men his limited calibre is very soon evident. In the present state of microscopy particularly, it is highly important that all books bearing on it should be rigidly practical in their character, and that the utmost precision of language should be observed. Microscopists are still in the condition of explorers; and as the field of their research is wide, they want guide-books which give real information as to what has been made out, and available instructions and hints for future investigations. It is with this view that we have expressed the foregoing opinions of Dr. Beale's book; if our estimate of the value of microscopy were less exalted, we should be less jealous of its reputation, and less anxious to have its scientific character maintained.

